# Efficacy of lenalidomide in myelodysplastic/myeloproliferative neoplasms with ring sideroblasts and an extreme platelet count

**DOI:** 10.1002/ccr3.3026

**Published:** 2020-06-13

**Authors:** Marion Divoux, Alexia Plocque, Margaux Sevin, Laurent Voillat, Pierre Feugier, Agnès Guerci‐Bresler, Francois Girodon, Julien Broséus

**Affiliations:** ^1^ Université de Lorraine, CHRU‐Nancy, Service d'Hématologie Clinique, Pôle Spécialités Médicales, France Nancy France; ^2^ Haematology Laboratory University Hospital Dijon France; ^3^ Inserm U1231 University of Bourgogne Franche‐Comté Dijon France; ^4^ Haemato‐Oncology Department Hospital of Chalon‐sur‐Saône Chalon‐sur‐Saône France; ^5^ Université de Lorraine, CHRU‐Nancy, Service d'Hématologie Biologique, Pôle Laboratoires, France Nancy France

**Keywords:** lenalidomide, myelodysplastic syndrome, myelodysplastic/myeloproliferative neoplasms with ring sideroblasts and thrombosis, myeloproliferative syndrome, thrombocytosis

## Abstract

Lenalidomide is efficient in reducing red blood cell transfusion dependency and markedly lowering platelet counts in MDS/MPN‐RS‐T in the context of major platelet counts.

## Graphical abstract

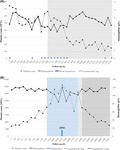

## 1 INTRODUCTION

Myelodysplastic/myeloproliferative neoplasms (MDS/MPN) with ring sideroblasts and thrombosis (MDS/MPN‐RS‐T), previously known as refractory anemia with ring sideroblasts and thrombocytosis (RARS‐T), are rare overlapping syndromes associating the dysplastic features of myelodysplastic syndromes with ring sideroblasts (MDS‐RS, previously known as refractory anemia with ring sideroblasts) and the myeloproliferative features of essential thrombocythemia (ET).^1^ MDS/MPN‐RS‐T present with clinical, biological, and prognostic features that differ from those of MDS‐RS and ET.^2^ Moreover, MDS/MPN‐RS‐T are characterized by a particular mutational pattern associating: (a) genomic abnormalities responsible for the myeloproliferative part, such as *JAK2^V617F^* mutations (40%‐50% of cases) or less frequently mutations in exon 10 of *MPL* (myeloproliferative leukemia) or in exon 9 of *CALR* (calreticulin) and (b) a high rate of splicing factor 3B subunit 1 (*SF3B1*) mutations, responsible for the myelodysplastic component of the disease.^2^, ^3^, ^4^, ^5^, ^6^ Thus, MDS/MPN‐RS‐T is now considered as an independent entity.^1^

The risk of thrombosis is higher in MDS/MPN‐RS‐T than in MDS‐RS patients without a high platelet count and this thrombotic risk often leads clinicians to use cytoreductive agents known to reduce the platelet count in almost 33% of cases.^2^ However, the use of cytoreductive agents is frequently interrupted, due to the worsening of cytopenia, especially anemia. The management and treatment of this disease are currently based on thrombosis risk stratification. When platelet count is <1000 × 10^9^/L, Patnaik and Tefferi^7^ recommend stratifying patients according to two thrombosis risk factors: age > 60 years and prior arterial or venous thrombosis. Patients with no risk factors should be treated with low‐dose aspirin or observation alone in *JAK2^V617F^*‐negative diseases with absence of thrombotic risk factors. Patients with 1 or 2 risk factors should be treated with low‐dose aspirin. Cytoreductive therapy with hydroxyurea is only recommended in case of high thrombotic risk. Lenalidomide is an immunomodulatory agent frequently used in low‐risk myelodysplastic syndromes.^8^ In this context, it is considered as third‐line treatment, in case of hydroxyurea failure associated with anemia. In case of a high platelet count >1000 × 10^9^/L, aspirin may exacerbate bleeding, cytoreductive therapy often worsens anemia and lenalidomide use is not suggested. Lenalidomide has been tested in published MDS/MPN‐RS‐T cases, with conflicting results.^9^, ^10^, ^11^, ^12^, ^13^, ^14^, ^15^ Here, we report our experience using lenalidomide on two patients with *JAK2* ^V617F^‐negative MDS/MPN‐RS‐T, one of them presenting with a major thrombocytosis.

## 2 CASES HISTORY

**Figure 1:**
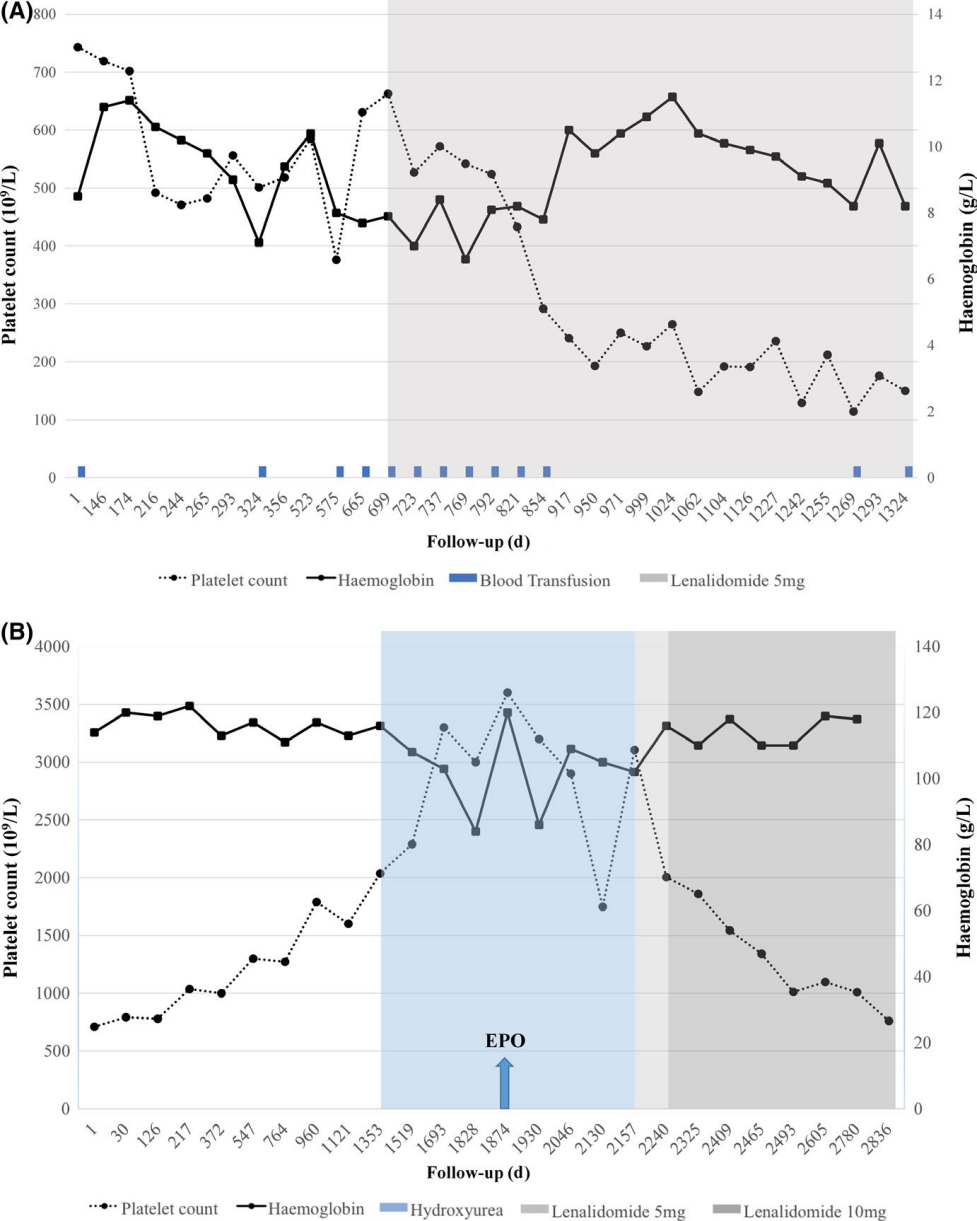
Course of blood counts for the two patients treated with lenalidomide: A, Patient 1; B, Patient 2. The left y‐axis represents the platelet counts (10^9^/L), and the right y‐axis represents hemoglobin level (g/L). The x‐axis represents the follow‐up (in days)

The first patient was a 78‐year‐old woman (Patient 1, Table 1), with a complete blood count (CBC) showing hemoglobin level at 85 g/L, persistent thrombocytosis (platelet count: 743 × 10^9^/L) and a leukocyte count at 6.4 × 10^9^/L. The bone marrow aspirates revealed erythroid hyperplasia with myelodysplastic features and 64% ring sideroblasts, no blast cells, associated with atypical megakaryocytes, leading to the diagnosis of MDS/MPN‐RS‐T, according to the revised 2016 world health organization classification's criteria.^1^ Bone marrow cytogenetics showed a normal 46XX karyotype. A Lys700Glu *SF3B1* mutation was noted, without *JAK2* ^V617F^, *MPL* exon 10 or *CALR* mutations. Blood transfusions were performed for one year but, in order to avoid relying on red blood cell transfusions, a treatment with lenalidomide (5 mg daily, 21/28 days) was started resulting in the decrease of platelet count from 686 × 10^9^/L (start of treatment) to 150 × 10^9^/L, associated with an improvement in hemoglobin levels from 80 to 100 g/L over the first 28 weeks of lenalidomide treatment. Along these 28 weeks after the beginning of lenalidomide, only 6 red blood cells (RBC) units were transfused (Figure 1A), and no transfusion was required in the subsequent 47 weeks since the hemoglobin level was above 90 g/L. However, due to the subsequent decrease in the hemoglobin level, RBC transfusions were later reinitiated, but with a lower frequency (2 units of RBC every 2 months), in association with lenalidomide. In other words, over the 20 months of treatment with lenalidomide, the RBC requirements were drastically reduced. However, grade IV neutropenia was observed (granulocytes: 0.5 × 10^9^/L), without any infectious disease, but leading to treatment stop.

**Table 1:** Biological characteristics at diagnosis of the two MDS/MPN‐RS‐T patients treated with lenalidomide

	Patient 1	Patient 2
Age (y)	78	58
Sex	F	F
Hb level (g/L)	85	114
MCV (fL)	92	97
Platelet count (10^9^/L)	743	710
Leukocytes (10^9^/L)	6.4	7.4
Ring sideroblasts (%)	64	24
Erythroid dysplasia	Yes	Yes
Megakaryocytic dysplasia	Marked	Marked
Excess of blasts	No	No
Karyotype	Normal	Normal
*SF3B1*	Mutated	N/A
*JAK2^V617F^*	No	No
*MPL^W515K/L^*	No	N/A
*CALR*	Unmutated	N/A
First treatment	RBC transfusions	Watch and wait
Evolution after 1st treatment	Increased transfusion dependency	Marked increase in the platelet count: 2000 × 10^9^/L
Second treatment	Lenalidomide 5 mg daily 21 d/28	Hydroxyurea 500 then 1000 mg/d
Evolution after 2nd treatment	Normal platelet count Hb increase to 100 g/L Transfusion frequency reduced	Adverse effects on hemoglobin levels. Irregular elevated platelet counts ranging from 1700 × 10^9^/L to 3622 × 10^9^/L. The decision to stop hydroxyurea and to start lenalidomide was made after a new increase in platelet count at 3106 × 10^9^/L. EPO was maintained once a week.
Third treatment	Not applicable	Lenalidomide 5 then 10 mg daily 21 d/28
Evolution after 3rd treatment	Not applicable	Major decrease in platelet count Subnormal Hb levels: 118 g/L EPO every 2 wk

Abbreviations: EPO, erythropoietin; F, female; Hb, hemoglobin; M, male; MCV, mean corpuscular volume; N, normal; N/A, nonavailable; RBC, red blood cells; y, years.

The second patient was a 58‐year‐old woman (Patient 2, Table 1) whose initial CBC showed hemoglobin 114 g/L, platelet count 710 × 10^9^/L, leukocytes 7.4 × 10^9^/L. Bone marrow aspirate showed erythroid hyperplasia with myelodysplastic features, no excess of blasts and 24% ring sideroblasts associated with atypical megakaryocytes, leading to the diagnosis of MDS/MPN‐RS‐T.^1^ Bone marrow cytogenetics showed a normal karyotype. A watch‐and‐wait strategy was initiated, but due to a marked increase in the platelet count > 2000 × 10^9^/L, hydroxyurea (500 mg/d and then 1000 mg/d) was started. It worsened the anemia to 108 g/L and then to 84 g/L, leading to a weekly use of erythropoietin. After 18 months, hydroxyurea was stopped due to (a) its adverse effects on hemoglobin level and (b) the observation of irregular elevated platelet counts ranging from 1700 × 10^9^/L to 3622 × 10^9^/L. After a drop of platelet count at 1700 × 10^9^/L, a new increase was observed at 3106 × 10^9^/L leading to the start of a lenalidomide treatment (5 mg daily, 21/28 days), secondarily increased to 10 mg daily (21/28 days) since very well tolerated. A marked decrease in the platelet count from 3106 to 760 × 10^9^/L was noted, while hemoglobin level raised up from 102 to 118 g/L (Figure 1B). However, for this patient, erythropoietin treatment was maintained every 2 weeks in combination with lenalidomide. Four years after the beginning of lenalidomide therapy, platelet count remains stable around 750 × 10^9^/L. Neither adverse effects, nor thrombosis or bleeding occurred.

Informed consent for publication was obtained from both patients.

## 3 DISCUSSION

In conclusion, this two‐case experiment with more than 3‐year follow‐up shows the efficacy of lenalidomide in normalizing (Patient 1) or markedly reducing (Patient 2) the platelet count and allowing independency from RBC transfusion in MDS/MPN‐RS‐T. To our best knowledge, ten MDS/MPN‐RS‐T cases have been published so far (Table 2).^9^, ^10^, ^11^, ^12^, ^13^, ^14^, ^15^ The efficacy of lenalidomide was constant in early stages, except for one case of advanced disease with rapid evolution in myelofibrosis and bone marrow failure.^10^ Efficacy of lenalidomide in reducing platelet count has been observed in 3 published cases (Table 2, patients 1, 7, and 10),^9^, ^14^, ^15^ in the context of moderately elevated platelet counts.

**Table 2:** Summary of data of the literature regarding MDS/MPN‐RS‐T case reports

Patient	Age (y)	Gender	Hb (g/L)	MCV (fL)	Platelet count (10^9^/L)	Leukocyte count (10^9^/L)	Ring sideroblasts (%)	Erythroid dysplasia	Megakaryocytic dysplasia	Karyotype	*JAK2^V617F^*	*MPL^W515K/L^*	*CALR*	*SF3B1*	First treatment	Evolution after first treatment	Second treatment	Evolution after second treatment	Third treatment	Evolution after third treatment	Citation
1	81	F	79	108	1677	10	86	Yes	Atypical megakaryocytes	Normal	Yes (qPCR) 19% load	N/A	N/A	N/A	EPO	EPO only temporally successful After 2 y: chronic pulmonary embolism	Hydroxyurea 500 mg 3×/d 2 wk	Reduced platelet count Transfusion dependency	Lenalidomide 5 mg daily	Platelets: 100 × 10^9^/L Transfusion independent Hb almost normalized *JAK2V617F* burden 0.8%	^9^
2	60	M	69	88	1592	12.7	98	Yes	Marked Hyperlobulated nuclei	Normal	Yes (qPCR) 32,3% load	N/A	N/A	N/A	Pyridoxine Anabolic steroids	Transfusion need	Lenalidomide 10 mg daily	3 RBC in 6 mo Plt: 680 × 10^9^/L *JAK2^V617F^* burden unchanged	N/A	N/A	^9^
3	47	F	112	N/A	700	N/A	25‐45	Yes		Normal	Yes	N/A	N/A	N/A	Hydroxyurea	Minor reduction of spleen size Worsening of anemia, RBC transfusion	Lenalidomide 10 mg daily 21 d/28	Pancytopenia, Increased transfusion requirement Grade 3 BM fibrosis	Allogeneic SCT	Graft lost Transfusion dependency Clonal evolution Death due to sepsis	^10^
4	84	F	77	N	1515	N/A	90	Yes	Numerous atypical megakaryocytes Hypolobulated nuclei	5q‐ (1 mitosis) Not confirmed by FISH	Yes 22% load	N/A	N/A	Unmutated	Transfusion Lenalidomide 10 mg daily	Platelets: 281 × 10^9^/L Transfusion independency BM normalization *JAK2^V617F^* burden <2%	N/A	N/A	N/A	N/A	^11^
5	39	F	82	122	1024	5.66	44	Yes	Highly atypical megakaryocytes Hyperlobulated nuclei	t(2;3)(p23;q29)	No (RT‐qPCR)	No	N/A	N/A	Hydroxyurea 1000 mg/d α‐interferon 3 M units 2×/wk	Reduced platelet count but worse anemia Transfusion dependence (4 units RBC/4 wk)	Hydroxyurea 500 mg 1×/d Pyridoxine Steroids EPO	Failure	Lenalidomide 5 mg daily 7 mo	Platelet drop 363 × 10^9^/L Hemoglobin: 90 g/L Transfusion independent Normal BM	^12^
6	58	M	98	N/A	1163	N/A	30	Yes	Large hyperlobulated nuclei	Normal FISH neg.	Yes (AS‐PCR)	N/A	N/A	N/A	Hydroxyurea 500 mg daily	Mild decrease of hemoglobin 83 g/L without efficacy	+ steroids EPO 40 000 units weekly	Inefficacy Transfusion dependency	Lenalidomide 10 mg daily 21 d/28	Transfusion independent; Hb > 9 g/dL Plt < 600 × 10^9^/L	^13^
7	68	F	61	N/A	1257	N/A	Positive	Yes	Megakaryocytic hyperplasia	Normal	No	No	Mutated	Mutated	Transfusion Iron supplementation	Symptoms improvement Thrombocytosis persistence Transfusion dependency	Lenalidomide 10 mg daily	Platelets: 497 × 10^9^/L Lenalidomide stopped because of severe nausea and anorexia. Platelets: 856 × 10^9^/L	Lenalidomide 5 mg daily	No toxicity Platelets: 351 × 10^9^/L Hemoglobin: 133 g/L No adverse events	^14^
8	49	M	107	93	935	9.2	65	Yes	Yes	Normal	Yes, allele burden 74%	No	No	Yes, allele burden 46%	Lenalidomide 10 mg daily	Decrease of platelet count to 585 × 10^9^/L. Stop lenalidomide after 8 mo due to loss of response	N/A	N/A	N/A	N/A	^15^
9	73	M	67	93.8	669	7.7	25	Yes	Yes	Normal	Yes, allele burden 40%	No	No	Yes, allele burden 25%	EPO	Transfusion dependency	Lenalidomide 10 mg daily	Transfusion independency Platelet count lowered to 470 × 10^9^/L Stop after 17 mo due to loss of response	N/A	N/A	^15^
10	85	F	68	88	1203	3	45	N/A	N/A	Normal	No	No	No	Yes, allele burden 44%	EPO, hydroxyurea, anagrelide	Transfusion dependency Suboptimal response	Lenalidomide 5 mg daily	Platelet count decrease: 558 × 10^9^/L Transfusion dependence	N/A	N/A	^15^
11	78	F	85	92	743	6.4	64	Yes	Marked	Normal	No	No	Unmutated	Mutated	Transfusion 1 y	Increased transfusion dependency	Lenalidomide 5 mg daily 21 d/28	Platelets: 150 × 10^9^/L Hb:80‐100 g/L – RBC requirement drastically reduced	N/A	N/A	Current work
12	58	F	114	97	710	7.4	24	Yes	Marked	Normal	No	N/A	N/A	N/A	Watch and wait	Marked increase in the platelet count: 2000 × 10^9^/L	Hydroxyurea 500 mg/d	Platelet count 3106 × 10^9^/L Hemoglobin: 84 g/L EPO 1×/wk Stop hydroxyurea	Lenalidomide 5 then 10 mg daily 21 d/28	Platelets: 760 × 10^9^/L Hemoglobin: 118 g/L EPO maintained every 2 wk	Current work

Abbreviations: AS‐PCR, allele‐specific polymerase chain reaction; BM, bone marrow; EPO, erythropoietin; F, female; Hb, hemoglobin; M, male; MCV, mean corpuscular volume; N, normal; N/A, non‐available; Plt, platelets; RBC, red blood cells; SCT, stem cell transplantation; y, years.

Our second case is striking, since it shows that lenalidomide induced a significant decrease in platelet count, even starting from very high counts (up to 3106 × 10^9^/L), along with an increase in hemoglobin level. So far, the efficacy of lenalidomide has never been shown in MDS/MPN‐RS‐T with such a high platelet count. It highlights the interest of lenalidomide as an alternative treatment for MDS/MPN‐RS‐T, including when they present with a major platelet count. Prospective trials are still needed to confirm those encouraging results, but because of rarity of the disease, such trials are very difficult to perform.

## CONFLICT OF INTEREST

None declared.

## AUTHOR CONTRIBUTIONS

DM: collected data on clinical and biological parameters, analyzed data, produced the figure, and wrote the manuscript. PA: collected data on clinical and biological parameters. SM: collected data on clinical and biological parameters. VL: collected data on clinical and biological parameters. FP: collected data on clinical and biological parameters. G‐BA: collected data on clinical and biological parameters. GF: analyzed data, collected data on clinical and biological parameters, and wrote the manuscript. BJ: analyzed data, collected data on clinical and biological parameters, and wrote the manuscript.
